# Supplementary material for: Effect of Spirulina platensis Supplementation on Carcass Characteristics, Fatty Acid Profile, and Meat Quality of Omani Goats
Source: Animals (Basel). 2023 Sep 20;13(18):2976. doi: 10.3390/ani13182976 (PMC10525089; doi:10.3390/ani13182976)
Supplement: Supplementary file 1 [file animals-13-02976-s001.zip › animals-2537467-supplementary.pdf]

**Table S1.** The effects of SP on final live body weight, hot carcass weight (HCWT), cold carcass weight (CCWT) and dressing-out%, carcass linear dimensions and major cut weights (least square means ± standard errors) of Jabbali and Sahrawi Omani goat breeds.

| **Parameters** | **Jabbali** | | | | **Sahrawi** | | | |
|---|---|---|---|---|---|---|---|---|
| | **CON** | **T1** | **T2** | ***P* value** | **CON** | **T1** | **T2** | ***P* value** |
| Final Live BW (kg) | 25.33±1.27 | 25.77±0.94 | 26.57±1.09 | 0.458 | 21.03±1.02 | 23.40±0.56 | 22.73±1.47 | 0.160 |
| HCWT (kg) | 11.09±0.35 | 11.60±0.35 | 12.26±0.87 | 0.107 | 9.96±0.78 | 10.84±0.18 | 10.41±0.62 | 0.385 |
| CCWT (kg) | 10.88±0.34 | 11.39±0.34 | 12.05±0.87 | 0.137 | 9.70±0.71 | 10.65±0.17 | 10.20±0.61 | 0.423 |
| Dressing-out (%)[1] | 43.87±1.22 | 45.20±2.75 | 46.06±1.62 | 0.785 | 47.29±2.38 | 46.34±0.39 | 45.95±2.25 | 0.698 |
| Gigwt (cm)[2] | 13.07±0.24 | 12.57±0.58 | 12.70±0.32 | 0.550 | 12.20±0.40 | 12.17±0.32 | 12.20±0.72 | 0.887 |
| Wtsh (cm)[3] | 17.33±0.94 | 16.10±1.29 | 18.20±0.21 | 0.480 | 15.37±0.43 | 15.40±0.29 | 16.67±0.58 | 0.337 |
| Msw (cm)[4] | 13.10±0.30 | 12.77±0.24 | 12.83±0.03 | 0.474 | 11.57±0.34 | 12.17±0.38 | 12.13±0.50 | 0.648 |
| Leg Length (cm) | 17.67±0.27 | 17.63±0.39 | 17.90±0.35 | 0.598 | 17.40$^{ab}$±0.53 | 18.45$^{a}$±1.49 | 16.60$^{b}$±0.29 | 0.025 |
| Body Length (cm) | 39.57±0.53 | 39.73±0.73 | 39.50±0.55 | 0.272 | 39.10$^{b}$±0.81 | 43.30$^{a}$±2.87 | 38.27$^{b}$±0.52 | 0.045 |
| DepSst (cm)[5] | 24.13±0.13 | 24.47±0.74 | 24.40±0.42 | 0.565 | 24.23±0.69 | 23.80±0.65 | 23.45±0.70 | 0.097 |
| Shol Weight (kg) | 4.76±0.25 | 5.01±0.21 | 5.56±0.38 | 0.151 | 4.43±0.43 | 4.63±0.16 | 4.62±0.39 | 0.629 |
| Rack Weight (kg) | 1.51±0.03 | 1.71±0.10 | 1.58±0.13 | 0.271 | 1.22$^{b}$±0.04 | 1.54$^{a}$±0.03 | 1.42$^{ab}$±0.12 | 0.272 |
| Loin Weight (kg) | 1.01$^{ab}$±0.08 | 1.06$^{a}$±0.05 | 0.98$^{b}$±0.06 | 0.019 | 0.83±0.05 | 1.01±0.06 | 0.88±0.08 | 0.421 |
| Leg Weight (kg) | 3.59±0.14 | 3.59±0.09 | 3.90±0.31 | 0.924 | 3.31±0.29 | 3.43±0.03 | 3.28±0.14 | 0.311 |

[1] Based on empty body weight, [2] Gigwt: gigot width, [3]Wtsh: Width behind shoulder, [4]Msw: Maximum Shoulder width and [5]DepSst: Depth from scapula to sternum. Means with different letters within the same row were significantly different ($P$<0.05).

**Table S2.** The effect of SP on non-carcass component (least square means ± standard errors) of Jabbali and Sahrawi Omani goat breeds.

| **Parameters[1]** | **Jabbali** | | | | **Sahrawi** | | | |
|---|---|---|---|---|---|---|---|---|
| | **CON** | **T1** | **T2** | **P value** | **CON** | **T1** | **T2** | **P value** |
| Head Weight (kg) | 1.63±0.02 | 1.79±0.04 | 1.78±0.12 | 0.170 | 1.48±0.09 | 1.73±0.03 | 1.57±0.08 | 0.338 |
| Feet Weight (g/kg) | 0.78±0.04 | 0.68±0.03 | 0.70±0.04 | 0.316 | 0.56±0.02 | 0.59±0.03 | 0.60±0.04 | 0.599 |
| Rumen full Weight (kg) | 4.90±0.56 | 5.00±0.95 | 5.37±0.27 | 0.786 | 3.67±0.34 | 4.37±0.18 | 4.32±0.68 | 0.926 |
| Rumen empty Weight (kg) | 1.98±0.16 | 2.13±0.15 | 2.25±0.13 | 0.248 | 1.54±0.05 | 1.69±0.17 | 1.86±0.17 | 0.716 |
| Lung Trachea Weight (g/kg) | 0.42±0.15 | 0.26±0.01 | 0.26±0.01 | 0.543 | 0.38±0.09 | 0.27±0.01 | 0.35±0.09 | 0.838 |
| Heart Weight (g/kg) | 0.36$^{a}$±0.27 | 0.09$^{b}$±0.01 | 0.09$^{b}$±0.01 | 0.667 | 0.09±0.00 | 0.09±0.01 | 0.25±0.17 | 0.834 |
| Spleen Weight (g/kg) | 0.05±0.01 | 0.05±0.00 | 0.05±0.00 | 0.977 | 0.05±0.00 | 0.06±0.01 | 0.06±0.01 | 0.216 |
| Liver Weight (g/kg) | 0.45±0.04 | 0.51±0.04 | 0.49±0.03 | 0.317 | 0.35±0.00 | 0.45±0.05 | 0.40±0.04 | 0.815 |
| Omental Fat Weight (g/kg) | 0.23$^{b}$±0.02 | 0.20$^{b}$±0.01 | 0.34$^{a}$±0.02 | 0.151 | 0.15$^{b}$±0.02 | 0.25$^{a}$±0.05 | 0.20$^{ab}$±0.04 | 0.711 |
| Kidney Weight (g/kg) | 0.29$^{a}$±0.21 | 0.06$^{b}$±0.01 | 0.06$^{b}$±0.01 | 0.151 | 0.06±0.01 | 0.07±0.00 | 0.06±0.00 | 0.714 |
| Kidney Fat Weight (g/kg) | 0.06$^{b}$±0.02 | 0.09$^{ab}$±0.02 | 0.15$^{a}$±0.01 | 0.094 | 0.06$^{b}$±0.01 | 0.13$^{a}$±0.03 | 0.09$^{ab}$±0.02 | 0.448 |
| Mesfat Weight (g/kg) | 0.17±0.04 | 0.18±0.02 | 0.19±0.02 | 0.409 | 0.14±0.02 | 0.15±0.07 | 0.13±0.04 | 0.506 |
| Mesenteric fat (g/kg) | 0.17±0.01 | 0.16±0.02 | 0.23±0.03 | 0.173 | 0.10±0.00 | 0.14±0.04 | 0.10±0.01 | 0.526 |
| Skin (kg) | 2.37±0.29 | 2.23±0.10 | 2.23±0.17 | 0.352 | 1.69±0.22 | 1.84±0.03 | 1.77±0.19 | 0.806 |

[1] all parameters are measured by kilogram. Means with different letters within the same row were significantly different ($P$<0.05).

**Table S3.** Least square means ± standard errors of the SP effects on meat quality characteristics of Longissimus Dorsi in Jabbali and Sahrawi breeds.

| Parameters | Jabbali | | | | Sahrawi | | | |
|---|---|---|---|---|---|---|---|---|
| | CON | T1 | T2 | P value | CON | T1 | T2 | P value |
| Ultimate ph | 5.38±0.30 | 5.42±0.23 | 5.64±0.12 | 0.987 | 5.19$^{b}$±0.09 | 5.84$^{a}$±0.17 | 5.22$^{b}$±0.12 | 0.126 |
| Sarcomere length (lm) | 10.00±0.42 | 9.50±0.69 | 9.67±0.67 | 0.707 | 8.17±0.95 | 9.67±0.76 | 8.78±0.46 | 0.183 |
| EJ (drip loss) (g/cm$^2$)[1] | 19.67±6.63 | 21.63±3.71 | 22.20±3.27 | 0.078 | 29.43±4.11 | 20.93±1.14 | 27.10±1.08 | 0.293 |
| Cook loss (%) | 41.20±3.31 | 37.73±3.43 | 38.60±4.33 | 0.976 | 41.27±3.54 | 33.63±3.59 | 41.57±2.23 | 0.836 |
| WBV (kg)[2] | 5.50±1.71 | 4.13±1.01 | 5.93±0.83 | 0.268 | 4.80±1.30 | 4.90±1.13 | 6.17±0.58 | 0.528 |
| L* (lightness) | 44.79±0.97 | 44.63±0.43 | 47.54±1.56 | 0.195 | 45.00$^{ab}$±2.21 | 41.52$^{b}$±0.62 | 46.55$^{a}$±1.49 | 0.685 |
| a* (redness) | 21.31±1.41 | 22.27±0.88 | 22.58±1.15 | 0.787 | 19.49±2.16 | 23.26±1.09 | 21.14±0.99 | 0.536 |
| b* (yellowness) | 5.65±0.20 | 5.61±1.01 | 5.89±1.46 | 0.625 | 4.95±0.99 | 4.53±0.45 | 6.06±0.91 | 0.567 |

[1]EJ (Expressed juice) = water area (cm$^2$)/sample weight (g). [2] WBV = Warner–Bratzler values. Means with different letters within the same row were significantly different (*P*<0.05).

**Table S4.** Least square means ± standard errors of the SP effects on meat quality characteristics of *semitendinosus* muscles in Jabbali and Sahrawi breeds.

| Parameters | Jabbali | | | | Sahrawi | | | |
|---|---|---|---|---|---|---|---|---|
| | CON | T1 | T2 | P value | CON | T1 | T2 | P value |
| Ultimate Ph | 5.97±0.33 | 6.02±0.43 | 6.37±0.13 | 0.881 | 6.08$^{ab}$±0.24 | 6.30$^{a}$±0.06 | 5.73$^{b}$±0.08 | 0.725 |
| Sarcomere length (lm) | 6.39±0.20 | 5.83±0.35 | 5.83±0.10 | 0.611 | 6.72±0.97 | 7.06±0.82 | 6.72±1.06 | 0.889 |
| EJ (drip loss) (g/cm$^2$)[1] | 28.97±3.55 | 24.17±6.21 | 26.60±2.04 | 0.850 | 19.97±3.46 | 21.47±3.26 | 27.03±2.44 | 0.370 |
| Cook loss (%) | 48.57±5.66 | 37.40±8.31 | 36.87±7.33 | 0.655 | 45.33±7.51 | 34.73±2.86 | 44.33±3.92 | 0.301 |
| Tender (kg) | 3.57±0.88 | 3.77±1.05 | 2.50±0.20 | 0.546 | 2.87±0.33 | 2.7±0.44 | 4.27±0.35 | 0.748 |
| L* (lightness) | 51.65±2.72 | 54.68±1.81 | 51.50±0.84 | 0.684 | 46.50$^{ab}$±0.81 | 43.41$^{b}$±0.82 | 48.69$^{a}$±0.65 | 0.088 |
| a* (redness) | 19.31±1.20 | 18.02±0.78 | 19.86±0.51 | 0.993 | 20.97±0.41 | 20.81±0.80 | 20.73±0.46 | 0.720 |
| b* (yellowness) | 4.91±0.26 | 4.93±1.21 | 5.56±0.09 | 0.894 | 4.81±0.24 | 4.65±0.45 | 5.49±0.47 | 0.494 |

[1]EJ (Expressed juice) =water area (cm$^2$)/sample weight (g). Means with different letters within the same row were significantly different (*P*<0.05).

**Table S5.** The effects of SP on fatty acid profile (g/100 g FA), groups (g/100 g fat), ratios, and indexes of Longissimus dorsi of Omani goats.

| Fatty acid | Jabbali | | | | Sahrawi | | | |
|---|---|---|---|---|---|---|---|---|
| | CON | T1 | T2 | *P* value | CON | T1 | T2 | *P* value |
| C10:0 | 0.05±0.01 | 0.06±0.02 | 0.08±0.03 | 0.896 | 0.15±0.09 | 0.11±0.08 | 0.14±0.03 | 0.055 |
| C12:0 | 0.06±0.02 | 0.06±0.03 | 0.25±0.22 | 0.980 | 0.24±0.12 | 0.41±0.38 | 0.27±0.07 | 0.025 |
| C13:0 | 0.01±0.00 | 0.01±0.00 | 0.01±0.01 | 0.856 | 0.05±0.01 | 0.40±0.39 | 0.03±0.01 | 0.012 |
| C14:0 | 0.98±0.27 | 1.00±0.63 | 1.47±0.61 | 0.922 | 2.32$^{ab}$±0.63 | 1.17$^{b}$±0.85 | 3.52$^{a}$±0.87 | 0.020 |
| C15:0 | 0.25±0.07 | 0.26±0.18 | 0.28±0.06 | 0.867 | 0.66$^{a}$±0.19 | 0.07$^{b}$±0.01 | 0.98$^{a}$±0.26 | 0.000 |
| C16:0 | 6.93±1.01 | 6.94±3.12 | 11.08±3.89 | 0.952 | 11.23±2.52 | 10.53±6.74 | 16.67±3.24 | 0.121 |
| C17:0 | 0.90±0.20 | 1.00±0.65 | 0.96±0.13 | 0.966 | 1.91$^{a}$±0.54 | 0.49$^{b}$±0.23 | 2.52$^{a}$±0.59 | 0.000 |
| C18:0 | 6.82±0.89 | 7.64±4.02 | 8.69±1.15 | 0.892 | 11.36$^{a}$±2.87 | 6.11$^{b}$±2.48 | 16.14$^{a}$±3.18 | 0.001 |
| C20:0 | 0.23±0.14 | 0.22±0.13 | 0.27±0.12 | 0.660 | 0.28±0.11 | 2.34±2.23 | 0.62±0.03 | 0.241 |
| C24:0 | 0.06±0.02 | 0.09±0.02 | 0.06±0.02 | 0.003 | 0.08±0.02 | 0.11±0.02 | 0.10±0.01 | 0.007 |
| Sfa [1] | 16.19±2.46 | 17.10±8.71 | 23.04±5.76 | 0.943 | 28.04$^{ab}$±6.84 | 19.95$^{b}$±11.68 | 40.57$^{a}$±8.29 | 0.040 |

| | | | | | | | | |
|---|---|---|---|---|---|---|---|---|
| C13:1 | 0.02±0.00 | 0.06±0.04 | 0.02±0.00 | 0.980 | 0.05±0.02 | 0.89±0.88 | 0.06±0.02 | 0.005 |
| C14:1 | 0.08±0.02 | 0.07±0.05 | 0.09±0.02 | 0.692 | 0.21[a]±0.07 | 0.02[b]±0.01 | 0.31[a]±0.08 | 0.000 |
| C16:1 | 0.24[a]±0.07 | 0.09[b]±0.03 | 0.27[a]±0.06 | 0.000 | 0.33[b]±0.12 | 0.17[b]±0.07 | 0.70[a]±0.18 | 0.005 |
| C17:1 | 0.74±0.19 | 0.73±0.43 | 1.00±0.38 | 0.876 | 1.93[a]±0.56 | 0.24[b]±0.02 | 1.94[a]±0.44 | 0.000 |
| C16:1Cis9 | 1.34±0.32 | 1.73±1.10 | 1.31±0.17 | 0.954 | 2.30[b]±0.63 | 0.75[c]±0.33 | 4.54[a]±1.09 | 0.001 |
| C18:1Cisn9 | 14.22±2.16 | 14.57±6.33 | 19.90±4.89 | 0.906 | 25.81±6.40 | 19.42±12.13 | 30.23±5.44 | 0.027 |
| Mufa [2] | 16.63±2.75 | 17.25±7.90 | 22.58±4.97 | 0.914 | 30.63±7.61 | 21.47±13.37 | 37.79±7.18 | 0.024 |
| C18:2Cisn6 | 3.60±1.07 | 2.55±0.81 | 2.93±0.88 | 0.348 | 1.83[b]±0.56 | 4.64[a]±1.62 | 4.46[a]±0.76 | 0.000 |
| C20:3n6 | 0.15[a]±0.04 | 0.06[b]±0.00 | 0.05[b]±0.00 | 0.586 | 0.07±0.01 | 0.07±0.01 | 0.17±0.09 | 0.824 |
| C20:4n6 | 0.81±0.06 | 0.88±0.10 | 0.68±0.08 | 0.174 | 0.95[b]±0.14 | 2.16[a]±0.77 | 0.78[b]±0.10 | 0.001 |
| C22:4n6 | 0.11±0.01 | 0.09±0.02 | 0.12±0.02 | 0.757 | 0.18±0.04 | 0.13±0.01 | 0.12±0.02 | 0.001 |
| Pufa n-6[3] | 4.61±1.06 | 3.58±0.87 | 3.77±0.86 | 0.307 | 3.03[b]±0.56 | 6.95[a]±2.25 | 5.45[a]±0.77 | 0.000 |
| C18:3Cisn3 | 0.09±0.01 | 0.11±0.06 | 0.11±0.02 | 0.974 | 0.13[b]±0.04 | 0.07[b]±0.01 | 0.23[a]±0.04 | 0.019 |
| cis-5,8,11-Eicosatrienoic | 0.18±0.02 | 0.15±0.04 | 0.18±0.02 | 0.993 | 0.27±0.07 | 0.56±0.41 | 0.28±0.06 | 0.008 |
| Pufa n-3[4] | 0.26±0.02 | 0.25±0.09 | 0.23±0.03 | 0.957 | 0.40±0.09 | 0.62±0.40 | 0.51±0.09 | 0.018 |
| n-6/n-3 ratio | 18.81±5.60 | 21.52±6.21 | 22.07±6.44 | 0.207 | 14.60[ab]±3.91 | 21.98[a]±4.39 | 11.77[b]±1.20 | 0.001 |

[1] Sfa, Total saturated fatty acids; [2] Mufa, total monounsaturated fatty acids; [3] Pufa n-6, total omega-6 polyunsaturated fatty acids; [4] Pufa n-3, total omega-3 polyunsaturated fatty acids. Means with different letters within the same row were significantly different ($P<0.05$).

**Table S6**: Basal diet components mean fatty acid composition (% total FA).

| Type of feed | Spirulina | concentrate | Rohds |
|---|---|---|---|
| C10:0 | 0.042 | 0.020 | 0.061 |
| C12:0 | 0.025 | - | 0.097 |
| C13 | 0.338 | 0.150 | 0.083 |
| C14:0 | 0.268 | 0.083 | - |
| C15:0 | 0.046 | 0.026 | 0.026 |
| C16:0 | 28.544 | - | 1.539 |
| C16:1 *Cis* 9 | 6.587 | 0.078 | - |
| C17:0 | 0.307 | 0.048 | 0.035 |
| C17:1 | 0.387 | 0.016 | - |
| C18:2 Cis (n6) | 0.420 | - | - |
| C18:0 | 1.319 | 0.849 | 0.264 |
| C18:1 *Cis (n9*) | 4.163 | 6.952 | 0.254 |
| C18:2 Cis (n6) | 17.024 | 10.146 | 0.661 |
| C20:0 | 0.104 | 0.212 | 0.074 |
| C:18:3 *Cis*(n3) | 14.619 | 0.222 | 1.211 |
| C21:0 | 15.000 | - | 15.000 |
| C20:3 (n6) | 0.200 | 0.285 | - |
| C20:5 (n3) | 0.096 | - | - |
| C22:6 (n3) | 0.088 | - | - |
